# PREPAREDNESS AND NEEDS AMONG YOUNG, MIDDLE-AGED, AND OLDER ADULT LATINX CAREGIVERS

**DOI:** 10.1093/geroni/igad104.3102

**Published:** 2023-12-21

**Authors:** Karen Kopera-Frye, Refat Srejon

**Affiliations:** New Mexico State University, Las Cruces, New Mexico, United States; New Mexico State University, Las Cruces, New Mexico, United States

## Abstract

Taking care of one’s family is a primary obligation among Latinx communities. According to CDC’s Behavioral Risk Factor Surveillance System, 22.9% of New Mexican adults reported providing care to a friend or family member in the past 30 days; 10.4 % provided care to an individual with dementia. The southern Border region of New Mexico is remote and desert, with high rates of poverty and inaccessibility to health care and services. Caregivers may feel unprepared for caregiving, lack the knowledge and skills required to provide care, but struggle through because of the importance of La Familia. Adverse physical and psychological consequences have been found; yet those who report greater preparedness exhibit lower levels of strain. Seventy-six primarily female and healthy Latinx caregivers (age range 20-76 years, 30% young, 40% middle-aged, and 30% older adults) were surveyed on caregiving they provide, administered the Preparedness for Caregiving Scale, and asked what types of services are needed in their role. Results indicated the top three provided aspects of caregiving were (in descending order): Food, emotional support, and transportation. Caregivers reported a high degree of preparedness in taking care of the recipient’s physical needs, handling emergencies, and proving overall care. Needed resources included emotional support, financial assistance, and respite. Greater caregiving provision was significantly associated with better health (r =. 53, p = .09) and greater resource needs (r = .35, p <.01). Thus, level of preparedness is an important caregiving dimension to address, especially with culturally diverse communities having strong cultural caregiving norms.

